# Reciprocal regulation of miR‐206 and IL‐6/STAT3 pathway mediates IL6‐induced gefitinib resistance in EGFR‐mutant lung cancer cells

**DOI:** 10.1111/jcmm.14592

**Published:** 2019-09-10

**Authors:** Yanhua Yang, Wei Wang, Hong Chang, Zenglei Han, Xinjuan Yu, Tingguo Zhang

**Affiliations:** ^1^ Department of Pathology, Qilu Hospital Shandong University Jinan China; ^2^ Department of Pathology Qingdao Municipal Hospital Qingdao China; ^3^ Department of Pathology The Affiliated Hospital of Qingdao University Qingdao China; ^4^ Department of Pathology The Third People's Hospital of Qingdao Qingdao China; ^5^ Center Laboratory Qingdao Municipal Hospital Qingdao China; ^6^ Department of Pathology, School of Basic Medical Sciences Shandong University Jinan China

**Keywords:** gefitinib, IL‐6, miR‐206, STAT3

## Abstract

Persistently activated IL‐6/STAT3 pathway promotes acquired resistance to targeted therapy with epidermal growth factor receptor‐tyrosine kinase inhibitors (EGFR‐TKIs) in non–small‐cell lung cancer (NSCLC) treatment. miR‐206 has been verified to be dysregulated and plays as a negative regulator in lung cancer. However, whether miR‐206 may overcome IL6‐induced gefitinib resistance in EGFR‐mutant lung cancer remains elusive. In this study, we investigated the role of miR‐206 in IL6‐induced gefitinib‐resistant EGFR‐mutated lung cancer cell lines. We showed that forced miR‐206 expression restored gefitinib sensitivity in IL6‐induced gefitinib‐resistant EGFR‐mutant lung cancer cells by inhibiting IL6/JAK1/STAT3 pathway. Specifically, mechanistic investigations revealed that miR‐206 blocked IL‐6/STAT3 signalling via directly targeting the 3'‐UTR of intracellular IL‐6 messenger RNA. Moreover, IL‐6 induced miR‐206 down‐regulation by reducing the cropping process of primary miR‐206 (pri‐miR‐206) into the Drosha/DGCR8 complex. Taken together, our findings reveal a direct role of miR‐206 in regulating IL‐6/STAT3 pathway and contrarily activated IL‐6/STAT3 signalling mediates the miR‐206 maturation process in gefitinib‐resistant EGFR‐mutant lung cancer cells.

## 1 INTRODUCTION

Lung cancer is the leading cause of cancer‐related mortality worldwide, with non–small‐cell lung cancer (NSCLC) being the most common type.^1^ For this type of cancer, somatic mutations within the kinase domain of the epidermal growth factor receptor (EGFR) lead to altered downstream signalling by the receptor and appear to define a subset of NSCLC characterized by ‘oncogene addiction’ to the EGFR pathway, which displays dramatic responses to the small molecule tyrosine kinase inhibitors (TKIs).^2^ Despite the initial response to first‐generation EGFR‐TKIs, patients with NSCLCs harbouring EGFR mutations acquire resistance to these agents, with a median time to disease progression of approximately 12 months.^3^ The most common mechanism of resistance (50%‐60% of patients) is the acquisition of a secondary T790M mutation on exon 20, which results in increased affinity for adenosine triphosphate (ATP), causing resistance to competitive inhibition by first‐/second‐generation EGFR‐TKIs.^4^ In addition to somatic mutation, activation of phenotypic or histological transformation, like epithelial‐mesenchymal transition (EMT), and bypass signalling pathway which is driving survival of the bulk population are also the main mechanisms facilitating EGFR‐TKI resistance.^5^

STAT3 is a transcription factor that can promote oncogenesis, and it is commonly activated in cancer as well as in tumour‐associated myeloid cells, which is frequently activated by inflammatory cytokine interleukin (IL)‐6.^6^ Experimental evidence suggested that deregulation of STAT3 activation by IL‐6 correlates with pancreatic cancer,^7^ colitis‐associated cancer,^8^ breast cancer^9^ and hepatocellular carcinoma.^10^ Functionally, over‐activated IL‐6/STAT3 signalling can induce drug resistance.^11^, ^12^, ^13^ Previous reports have demonstrated that IL‐6 promoted the EMT of lung cancer cell^14^, ^15^, ^16^ and increased IL‐6 activated signal transducer and activator of transcription 3 (STAT3) signalling pathway in lung adenocarcinoma.^17^ Apparently, IL‐6/STAT3 signalling pathway may be implicated in EGFR‐TKI resistance. Yao et al^18^ and Li et al^19^ further confirmed that erlotinib‐resistant cells have become unleashed from their EGFR activity dependence and relied on IL‐6/STAT3‐mediated signalling for their survival and suppression of IL‐6/STAT3 pathway abrogated acquired EGFR‐TKI resistance, respectively. For this reason, combinative inhibition of EGFR and IL‐6/STAT3 pathway rather than blockade of EGFR alone might therefore be more effective in the treatment of lung cancer.

MicroRNAs (miRNA) are a family of small non‐coding RNAs that negatively regulate target gene expression at post‐transcriptional level.^20^ Specifically, evidence suggested that dysregulation of specific miRNAs may be involved in the acquisition of resistance to a number of cancer treatments, thereby modulating the sensitivity of cancer cells to such therapies.^21^ In lung cancer, most miRNAs affect EGFR‐TKI resistance by targeting c‐Met,^22^ PI3K/AKT,^23^, ^24^ apoptosis^25^ and EGFR expression,^26^, ^27^ although EGFR‐TKI‐treated lung cancer cells inherently engage a positive feedback activation of STAT3 upon EGFR inhibition.^28^ Several differentially expressed miRNAs were identified and found to regulate IL‐6/STAT3 signalling pathway repression of multiple cancers.^29^, ^30^, ^31^ However, it has not been determined whether certain miRNA is also involved in IL‐6/STAT3 signalling to regulate EGFR‐TKI sensitivity in lung cancer.

MiR‐206 was first discovered as a central regulator in the regulation of myogenesis, muscle development and muscle remodelling.^32^ Further study has revealed that deregulation of miR‐206 occurs in lung adenocarcinoma correlating with poor prognosis and survival.^33^ Chen et al have found miR‐206 regulates cisplatin resistance and EMT in human lung adenocarcinoma cells partly by targeting Met.^34^ In addition, miR‐206 plays a role, involved in the invasion and metastasis of lung cancer,^35^ in HGF‐induced gefitinib‐resistant human lung cancer cells through inhibition of c‐Met signalling and EMT.^36^ These promising studies point to the importance of further understanding the role of miR‐206 in drug resistance in lung cancer. Here, we investigated the role of miR‐206 in regulating IL‐6/STAT3 pathway and gefitinib resistance in lung cancer.

## 2 MATERIALS AND METHODS

### 2.1 Clinical tissues

Tissue and serum specimens were obtained from 37 patients (Table S1) and 14 healthy participants in Qilu Hospital between 2015 and 2018. The patients were diagnosed with NSCLC based on histopathological evaluation and treated with gefitinib (Iressa, Tocris Bioscience) for at least 6 months. No local or systemic treatment was conducted in these patients before surgery. All collected tissue and serum samples were immediately snap‐frozen in liquid nitrogen and stored at −80°C until use. The study was approved by the Research Ethics Committee of Qilu Hospital, China. Written informed consent was obtained from all patients.

### 2.2 Cell line culture

PC‐9 and HCC827 cells were purchased from the Committee on Type Culture Collection of Chinese Academy of Sciences PC‐9 cells were maintained in Dulbecco's modified Eagle's medium (DMEM; Gibco) and supplemented with 10% foetal bovine serum (FBS) and antibiotics and cultured at 37°C in humidified air with 5% CO_2_. HCC827 cells were cultured in RPMI 1640 (Gibco) containing 10% FBS and antibiotics and cultured at 37°C in humidified air with 5% CO_2_. Recombinant human IL‐6 was bought from R&D Systems.

### 2.3 RNA isolation and real‐time quantitative PCR

Total RNA was extracted from cell lines and frozen tumour specimens using Trizol reagent (Invitrogen) and treated with DNase I (Invitrogen). Complementary DNA synthesis was performed using PrimeScript^TM^ RT reagent Kit (RR037A, Takara) according to the manufacturer's instructions. SYBR^TM^ Green PCR Master Mix (4368577, Applied Biosystems) was used to transcribe cDNA and quantify gene expression. MiRNA was transcribed and quantified by All‐in‐One^TM^ miRNA qRT‐PCR Detection Kit (QP016, GeneCopoeia^TM^). The quantitative real‐time PCR was performed on Applied Biosystems™ 7500 Fast Dx Real‐Time PCR system (Applied Biosystems) with specific primers (Table S2) following the instructions of manufacturer. GAPDH or U6 was used as an endogenous control. Expression of miRNA and protein was normalized to U6 and GAPDH, respectively.

### 2.4 Measurement of IL‐6

IL‐6 levels in patients' and healthy participants' plasma samples were determined using an enzyme‐linked immunosorbent assay (ELISA) kit (R&D Systems). For intracellular IL‐6 assessment, cells were lysed in Trizol reagent (Invitrogen) and treated with DNase I (Invitrogen) for qRT‐PCR assay.

### 2.5 Cell viability assays

Cells were seeded at a density of 5 × 10^3^ cells per well in 96‐well culture plates. The next day, cells were treated with miRNA mimics at indicated gefitinib concentration in a final volume of 100 μL for 72 hours. 10 µL of CCK‐8 solution (Cell counting KIT‐8, Solarbio) was added into each well, and the absorbance at 450 nm was measured after incubation for 30 minutes at 37°C to reflect the number of viable cells. All experiments were conducted by Multiskan Sky (Thermo Fisher) in triplicate.

### 2.6 Cell apoptosis analysis

Cell apoptosis analyses were performed using Annexin V‐FITC/PI Apoptosis Detection Kit (DOJINDO). Cells were seeded in 6‐well plates at 1 × 10^6^ per well. The next day, cells were treated with 0.1 μmol/L gefitinib and/or miRNA mimics. 24 hours after transfection, cells were harvested and resuspended at 1 × 10^6^ in 100 μL volume. Cells were labelled with 5 μL Annexin V and 5 μL PI for 15 minutes in dark place. 400 μL of 1 Annexin V Binding Solution was added, and the samples were detected by flow cytometry within 1 hour.

### 2.7 Colony formation assays

PC‐9 and HCC827 were plated in 6‐well culture dishes at a density of 500 cells/well. Cells were treated with 0.1 μmol/L gefitinib and/or 20 nmol/L miR‐206 mimics for 7 days. Cells were stained with crystal violet on the plates. Cell colonies were photographed under an inverted microscope.

### 2.8 Co‐immunoprecipitation and Western blot

For co‐immunoprecipitation, cells were lysed using RIPA protein extraction reagent (Beyotime) supplemented with a protease inhibitor cocktail (Roche) and PMSF (Roche). The protein concentration was measured using the Bio‐Rad protein assay kit. The supernatants were collected and incubated with anti‐pStat3, Drosha at 4°C for 12 hours. Protein A Sepharose CL‐4B beads (GE) were incubated with the mixture at 4°C for 2 hours. Then, the beads were washed three times with RIPA buffer. The bound proteins were eluted with SDS‐PAGE loading buffer and used for Western blot. For Western blot assay, approximately 40 μg of protein extract was electrophoresed on a 10% SDS‐polyacrylamide gel electrophoresis (SDS‐PAGE) and then transferred onto 0.22‐μm nitrocellulose membrane (Sigma) and incubated with specific antibodies. The ECL chromogenic substrate was used to visualize the bands. The intensity of the protein bands was quantified by Quantity One software (Bio‐Rad ChemiDoc XRS). GAPDH was used as a control. Antibodies for Jak1, p‐Jak1, Stat3, p‐Stat3, Ago, IL‐6, Drosha, DGCR8 and GAPDH were purchased from Abcam.

### 2.9 Cell transfection

miRNA mimics or siRNA transfection was conducted with PEIpro (Polyplus) according to the manufacturer's instructions. Briefly, the cells were transfected with 20 nmol/L miRNA mimics or 50 nmol/L siRNA for 6 hours with PEIpro reagent and replaced with fresh growth medium. The next day, cells were treated with gefitinib for Western blot, qRT‐PCR or MTT assay. siRNA for IL‐6 was synthesized by Sangon Biotech, and the sequences were shown in Table S2.

### 2.10 Luciferase reporter assay

The 3′‐UTR of wild or mutant IL‐6 was synthesized by Sangon Biotech and inserted into pMiR‐Report firefly luciferase vector (GenePharma). pRL‐Tk Renilla luciferase reporter was used for luciferase assay normalization. PC‐9 cells were transfected with 50 ng wild‐type or mutant luciferase reporters and miR‐206 mimics or control, along with 10 ng Renilla luciferase vector with the PEIpro (Polyplus) reagent. After 48 hours, luciferase activity was detected by the Dual‐Luciferase Reporter Assay System (Promega) and relative luciferase activity was normalized to Renilla luciferase activity.

### 2.11 RNA‐ChIP assay

The cells were crosslinked and processed according to the RNA ChIP‐IT^®^ Magnetic Chromatin Immunoprecipitation Kit (Active Motif, 53024) protocol. Antibodies to Ago control IgG (Abcam) were used at 4 μg per 10 μg sheared chromatin. 20 μL sonicated but pre‐immunoprecipitated RNA from each sample was used as input control. ChIP results were analysed by qRT‐PCR. The primers locating the flank of pri‐miR‐197 stem‐loop were designed and shown in Table S2.

### 2.12 Statistical analysis

All data were presented as mean ± SD values or min to max values. Student's t test (two‐tailed) was performed to analyse the data from the experiments performed in triplicate, *P < .05, **P < .01, ***P < .001. For analysing the association of miR‐206 expression and serum IL‐6 levels, Spearman's correlation in Prism 7 was used with the *P* values indicated.

## 3 RESULTS

### 3.1 miR‐206 is dramatically down‐regulated and negatively correlated with IL‐6 in gefitinib‐resistant EGFR‐mutant lung carcinoma

**Figure 1:**
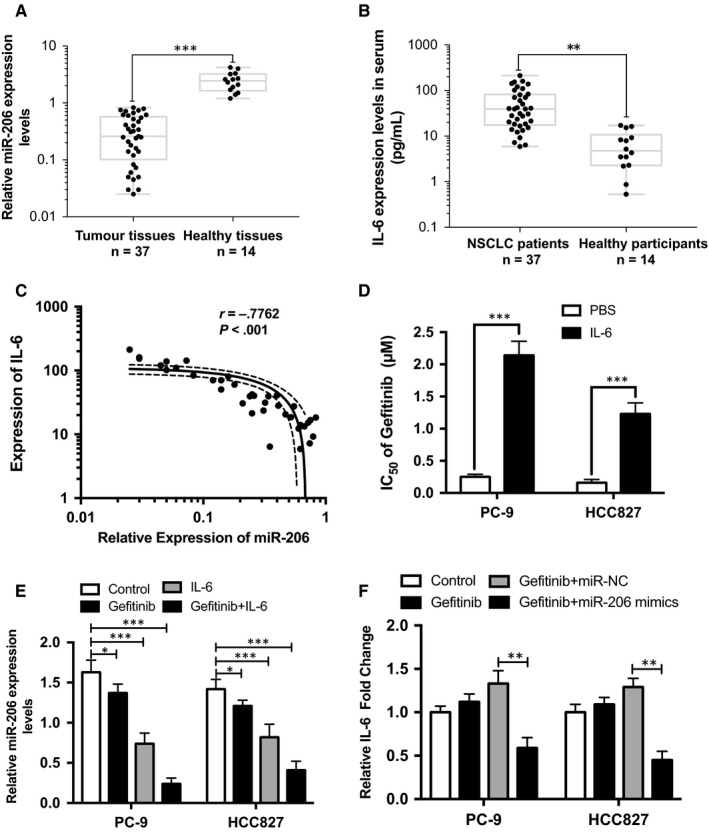
miR‐206 was dramatically down‐regulated and negatively correlated with IL‐6 in IL‐6‐induced gefitinib‐resistant EGFR‐mutant lung carcinoma. A, relative miR‐206 expression in gefitinib‐resistant patients and healthy participants. B, the levels of serum IL‐6 in gefitinib‐resistant patients and healthy participants. C, the association of miR‐206 expression and serum IL‐6 levels was determined by Spearman's correlation. D, IC_50_ of gefitinib in IL‐6‐treated EGFR‐mutant lung cancer cells. E, relative miR‐206 expression in IL‐6‐treated EGFR‐mutant lung cancer cells. F, the levels of IL‐6 mRNA in miR‐206‐treated EGFR‐mutant lung cancer cells. The min to max values and mean ± SD values are shown. *P < .05, **P < .01, ***P < .001

To determine whether miR‐206 is involved in IL‐6/STAT3 signalling to regulate gefitinib sensitivity in lung cancer, we evaluated the expression of miR‐206 and IL‐6 in 37 NSCLC patients harbouring EGFR mutations and 14 healthy participants as IL‐6 secreted by tumour cells was postulated as a potential mechanism for the primary resistance or low sensitivity to EGFR‐TKIs.^37^ The patients' backgrounds and clinical characteristics are listed in Table S1. The expression levels of miR‐206 were dramatically reduced in tumour tissues compared to healthy participants' normal lung tissues (Figure 1A), whereas the levels of serum IL‐6 were significantly increased in NSCLC patients (Figure 1B). Spearman's rank test showed a negative correlation between the expression of miR‐206 and that of IL‐6 (*r* = −.7762, *P* < .001, Figure 1C). In parallel, we adapted two EGFR‐mutant and TKI‐sensitive lung cancer cell lines, PC‐9 and HCC827, to IL‐6 and cultured for 72 hours to simulate the in vivo microenvironment. In accordance with prior study,^38^ activation of IL‐6 could induce resistance to EGFR inhibitor (Figure 1D). Surprisingly, we also found the reciprocal regulation of miR‐206 and IL‐6 in the gefitinib setting (Figure 1E,F). These data suggested that miR‐206 may be relevant to IL‐6 downstream signalling pathway in EGFR‐mutant lung cancer cells.

### 3.2 miR‐206 restores gefitinib sensitivity in IL6‐induced gefitinib‐resistant EGFR‐mutant lung cancer cells

**Figure 2:**
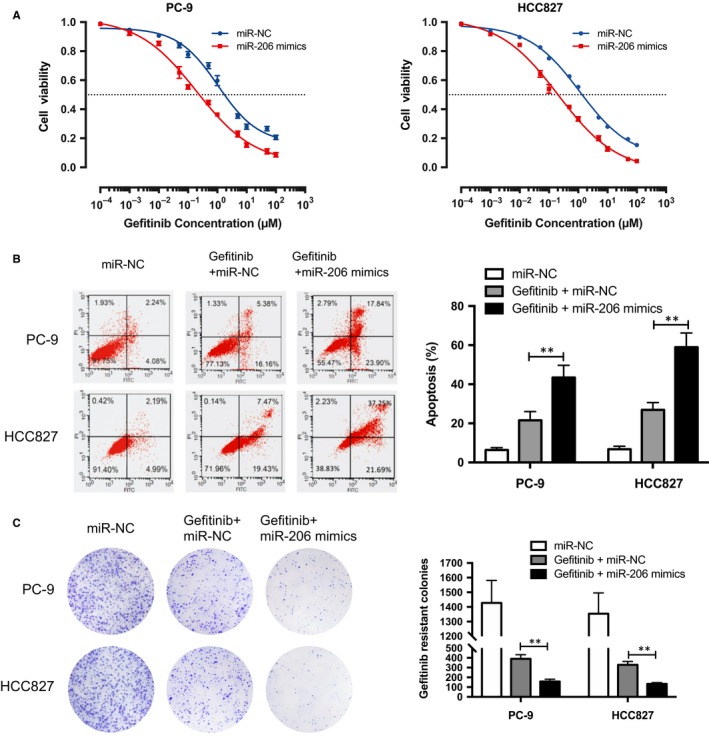
miR‐206 overcame IL‐6‐induced gefitinib resistance in PC‐9 and HCC827 cells. A, cells were treated with gefitinib for 24 h to measure viability by CCK‐8 assay. B, cells were treated with 0.1 μmol/L gefitinib and/or 20 nmol/L miR‐206 mimics for 6 h to measure apoptosis by flow cytometry. C, cells were treated with 0.1 μmol/L gefitinib and/or 20 nmol/L miR‐206 mimics for 7 d to measure gefitinib‐resistant colony formation. PC‐9 and HCC827 cells were cultured for 72 h with 10 ng/mL rhIL‐6 prior to gefitinib or mimics treatment. The mean ± SD values are shown. **P < .01

To investigate the functional importance of miR‐206 in IL6‐induced gefitinib‐resistant EGFR‐mutant lung cancer cells, IL‐6‐treated PC‐9 and HCC827 cells were transfected with miR‐206 mimics or negative control miRNA (miR‐NC). Forced expression of miR‐206 by miRNA mimics in IL‐6‐treated EGFR‐mutant cell lines significantly reduced their IL‐6 rendered gefitinib resistance as measured by cell viability assay (Figure 2A). Consistent with cell viability analysis, miR‐206 mimics dramatically accelerated apoptosis by almost twofold following gefitinib treatment (Figure 2B). Furthermore, to visualize the growth of IL‐6‐treated EGFR‐mutant cell lines, gefitinib‐resistant colonies were stained with crystal violet on the plates. As shown in Figure 2C, gefitinib‐resistant colonies were intensively decreased upon miR‐206 mimics treatment. These findings indicated that miR‐206 is a potential suppressor of IL6‐induced gefitinib resistance in PC‐9 and HCC827 cells.

### 3.3 miR‐206 inactivates IL‐6/JAK1/STAT3 pathway in IL6‐induced gefitinib‐resistant EGFR‐mutant lung cancer cells

**Figure 3:**
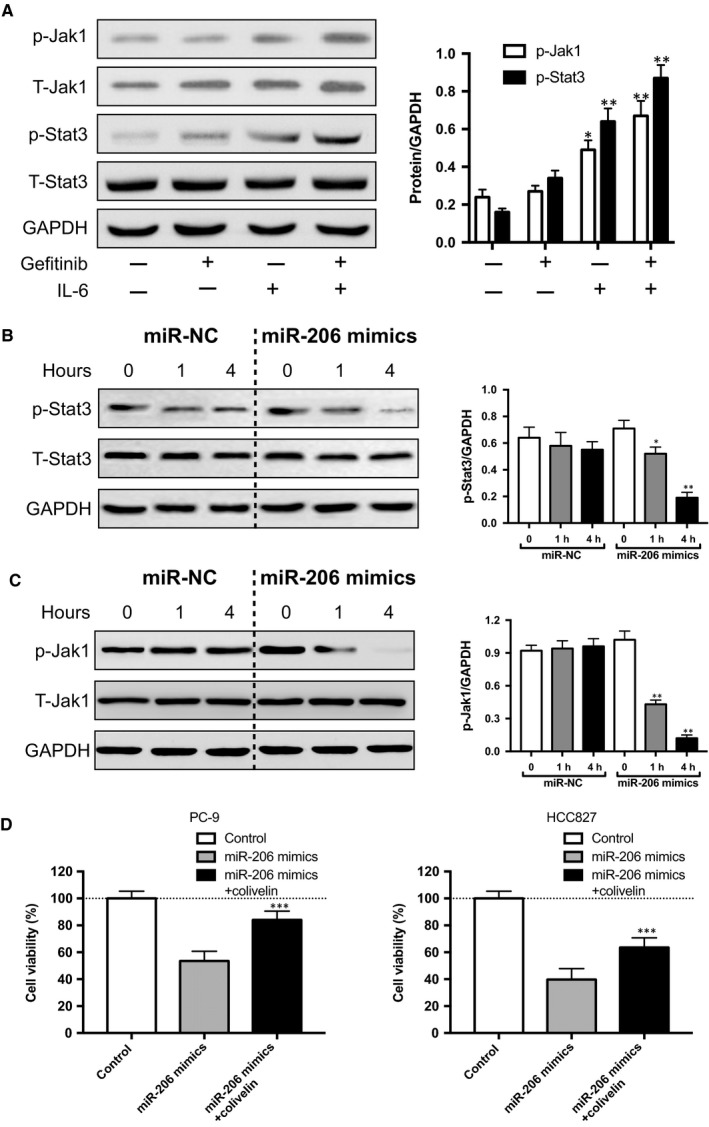
Forced miR‐206 expression blocked IL‐6/Jak1/Stat3 cascade. A, IL‐6‐activated Jak1‐Stat3 signalling was determined by Western blot. B‐C, miR‐206 mimics abolished IL‐6‐induced p‐Stat3 and p‐Jak1. PC‐9 cells were cultured for 72 h with 10 ng/mL rhIL‐6 prior to mimics treatment. D, colivelin significantly abrogated miR‐206 increased gefitinib sensitivity in PC‐9 and HCC827 cell; PC‐9 cells were transfected with 20 nmol/L miR‐206 mimics or 20 nmol/L miR‐206 mimics plus 50 nmol/L colivelin along with 0.1 μmol/L gefitinib treatment for 6 h to measure apoptosis by flow cytometry, the control group was only treated with 0.1 μmol/L gefitinib, and the cell viability was normalized to the control group. The min to max values and mean ± SD values are shown. *P < .05, **P < .01, ***P < .001

The significantly suppressive effect of miR‐206 on IL6‐induced gefitinib‐resistant EGFR‐mutant lung cancer cells prompted us to investigate its downstream signalling pathway. Previous reports have confirmed that IL‐6/JAK1/STAT3 pathway is the basic mechanism to promote gefitinib resistance lung cancer.^38^, ^39^ In comply with these reports, IL‐6 treatment activated the phosphorylation of JAK1 and STAT3, while left the total amount of JAK1 and STAT3 unchanged (Figure 3A). Nevertheless, forced expression of miR‐206 reduced the phosphorylated‐JAK1 (p‐JAK1) and p‐STAT3 (Figure 3B,C). Next, we examined whether STAT3 directly participated in miR‐206‐mediated gefitinib sensitivity by activating STAT3. We used colivelin, a STAT3 activator (Figure S1), which suppresses neuronal death by activating STAT3.^40^ The viability assay showed that addition of 50 nmol/L colivelin significantly abrogated miR‐206 increased gefitinib sensitivity (Figure 3D). Taken together, these results showed that miR‐206 mediated IL6‐induced gefitinib sensitivity by targeting JAK1/STAT3 pathway.

### 3.4 Intracellular IL‐6 is the direct target of miR‐206

**Figure 4:**
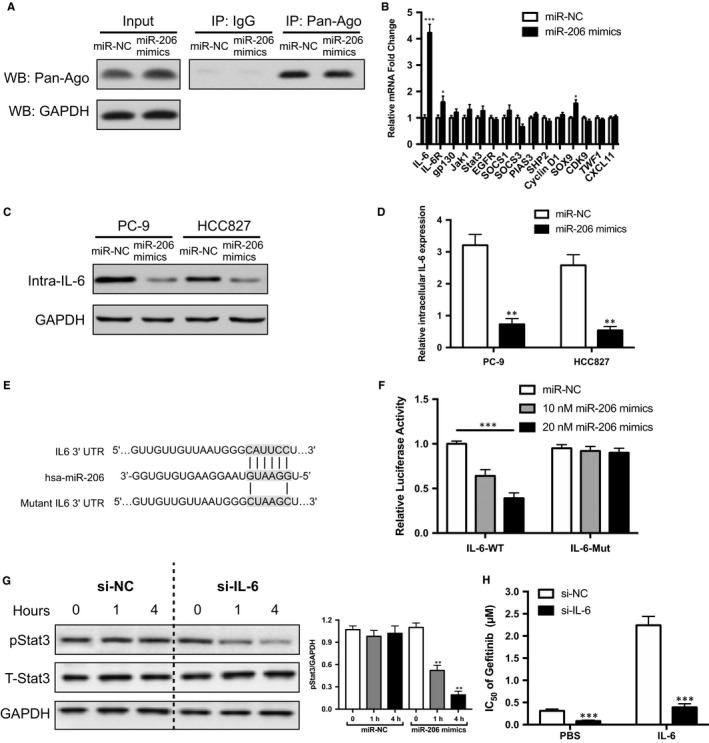
Intracellular IL‐6 was the direct target of miR‐206. A, Western blot was used to detect the Ago2‐RISC complex using the Ago antibody in PC‐9 cells with miR‐206 mimics or control; IgG was used as a negative control. B, RNA‐ChIP analysis was conducted to detect levels of mRNAs that bound with the Ago2‐RISC complex from PC‐9 cells transfecting with miR‐206 mimics or control for 4 h, as measured by qRT‐PCR; C, Western blot or D, qRT‐PCR was used to detect the protein or mRNA levels of intracellular IL‐6 in PC‐9 and HCC827 cells; PC‐9 and HCC827 cells were cultured for 72 h with 10 ng/mL rhIL‐6 prior to miR‐206 mimics or control treatment, and the cells collected for WB were washed five times to remove remnant membrane‐bounded rhIL‐6 with PBS before lysis. E, bioinformatics predicted and mutated miR‐206 binding sites with IL‐6. F, luciferase activity of the reporter construct containing the wild‐type or mutant 3' UTR of IL‐6 was measured after cotransfection with different amounts of miR‐206 mimics in PC9 cells. G, knockdown of intracellular IL‐6 abolished IL‐6‐induced p‐Stat3 in PC‐9 cell; PC‐9 cells were cultured for 72 h with 10 ng/mL rhIL‐6 prior to siRNA transfection. H, knockdown of intracellular IL‐6 decreased gefitinib resistance in PC‐9 cells. The mean ± SD values are shown. *P < .05, **P < .01, ***P < .001

The potent effects of miR‐206 in reducing the IL6‐induced gefitinib resistance in EGFR‐mutant lung cancer prompted us to explore the direct downstream effector of miR‐206. MiRNA exerts its influence by loading into an Argonaute (Ago) protein within the RNA‐induced silencing complex (RISC), which mediates repression of targets.^41^ Therefore, we employed RNA‐ChIP analysis to identify the mRNAs selectively enriched in the Ago2/RISC complex after miR‐206 overexpression by immunoprecipitating Ago protein (Figure 4A). A series of predicated mRNA were relative quantified by qRT‐PCR, which were either the main regulators of IL‐6/STAT3 pathway or previously identified targets of miR‐206. Among the 15 genes, mRNA of IL‐6 was detected with the most significant enrichment in miR‐206‐overexpressing IL‐6‐treated PC‐9 cells compared with the miR‐NC group (Figure 4B). Consistent with RNA‐ChIP analysis, the protein level of intracellular IL‐6 (Figure 4C) and mRNA expression (Figure 4D) were found to be decreased with miR‐206 mimics treatment in PC‐9 and HCC827 cells. Bioinformatics analysis by TargetScan and miRBase confirmed the putative binding site between IL‐6 and miR‐206 (Figure 4E). To further explore whether miR‐206 suppresses intracellular IL‐6 directly through the putative binding sites in the 3' UTR of intracellular IL‐6, a luciferase reporter was employed, in which the 3' UTR with wild‐type or mutated miR‐206 binding sites were embedded downstream of the dual‐luciferase reporter vector. Indeed, the luciferase expression was repressed by miR‐206 in a dose‐dependent manner in PC‐9 cells, whereas that with mutated 3' UTR was not altered significantly (Figure 4F). Moreover, knockdown of intracellular IL‐6 mRNA by siRNA reduced the activation of STAT3 (Figure S2 and Figure 4G) and decreased the IC_50_ of gefitinib in PC‐9 cells (Figure 4H). In sum, these data support that the intracellular IL‐6 serves as a direct target of miR‐206 in EGFR‐mutant lung cancer cells.

### 3.5 IL‐6 induces down‐regulation of miR‐206 by decreasing incorporation of pri‐miR‐206 into the Drosha/DGCR8 complex

**Figure 5:**
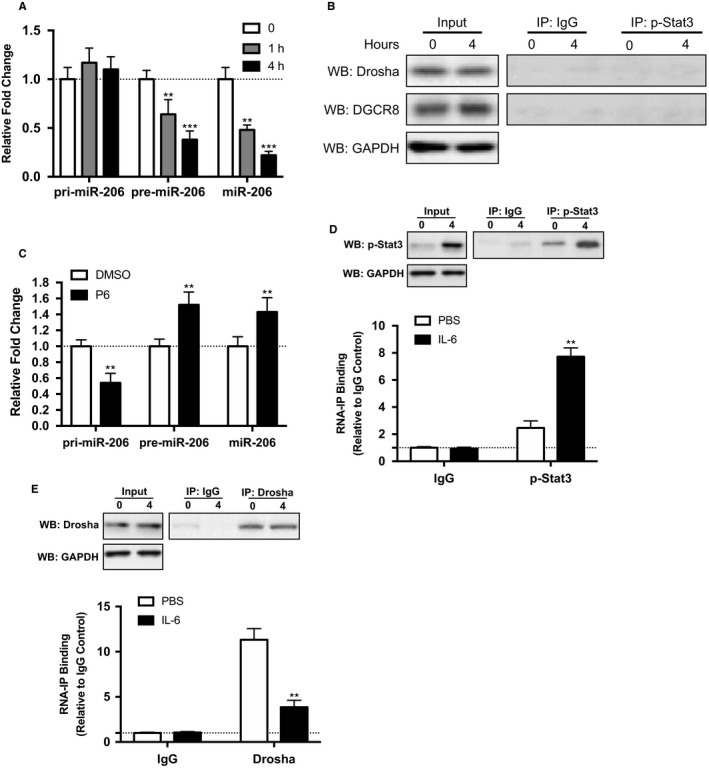
IL‐6‐induced p‐Stat3 interacted with Drosha/DGCR8 complexes and decreased incorporation of pri‐miR‐206 into the complex to hinder its processing. A, relative expression of pri‐miR‐206, pre‐miR‐206 and mature miR‐206 after 10 ng/mL rhIL‐6 treatment in PC‐9 cell. B, co‐immunoprecipitation of pStat3 was performed in PC‐9 cells by determination of protein levels of the associated Drosha and DGCR8 through Western blot. C, inhibition of pStat3 restored miR‐206 maturation pathway; the qRT‐PCR was performed 4 h after 5 nmol/L P6 or DMSO treatment in 10 ng/mL rhIL‐6 treated PC‐9 cell. D‐E, RNA‐ChIP was used to detect association between pri‐miR‐206 and p‐Stat3 or Drosha. The mean ± SD values are shown. **P < .01, ***P < .001

In Figure 1E, IL‐6 could induce down‐regulation of miR‐206, while the relative mechanism has not been elucidated. We first speculated that the biogenesis of miR‐206 may be the most direct way to modulate its expression by IL‐6. Hence, we measured the expression changes of pri‐miR‐206, pre‐miR‐206 and mature miR‐206 from PC‐9 cells. As shown in Figure 5A, stimulation with IL‐6 significantly reduced mature miR‐206 and pre‐miR‐206 expression while pri‐miR‐206 expression level remained stable, suggesting that processing of miR‐206 is regulated at a post‐transcriptional step likely through the action of the Drosha/DGCR8 complex. We next detected the Drosha/DGCR8 expression upon IL‐6 stimulation. However, IL‐6 stimulation did not induce up‐regulation of Drosha/DGCR8 protein (Figure 5B left). What's more, p‐STAT3, as the key downstream effector, exhibited no direct association with Drosha/DGCR8 (Figure 5B right). To examine the role of STAT3 in the post‐transcriptional regulation of miR‐206 maturation, we inhibited STAT3 by pyridone 6 (P6), pan‐JAK inhibitor (Figure S3). STAT3 inhibition abrogated the reduction of pre‐miR‐206 and miR‐206 and elevation of pri‐miR‐206 by IL‐6 (Figure 5C). The above data implied that p‐STAT3 may directly be associated with pri‐miR‐206, not Drosha/DGCR8 complex, to reduce its maturation process. To confirm this speculation, RNA‐ChIP analysis was performed on PC‐9 cells supplemented with IL‐6 or not, and an enhanced association of p‐STAT3 with pri‐miR‐206 was detected (Figure 5D). Further, the association between pri‐miR‐206 and Drosha was significantly reduced in PC‐9 cells cultured with IL‐6 (Figure 5E). Collectively, these data prove that p‐STAT3 specifically binds with pri‐miR‐206 to inhibit its recruitment into the Drosha/ DGCR8 complex.

## 4 DISCUSSION

Alterations in the EGFR itself, somatic mutation or ALK rearrangement, and activation of alternative signalling pathways have been shown to induce acquired TKI resistance in EGFR‐mutant NSCLC.^42^ In several studies, EGFR inhibition with TKI concurrently activated STAT3 signalling in EGFR‐mutant lung cancer cells,^28^, ^38^, ^43^ by which cancer cells could utilize an alternative signalling pathway to evade the drugs designed for ‘addition oncogene’ EGFR. Nonetheless, the direct cause of why TKI treatment over‐activated STAT3 for survival is unclear. Previous studies have demonstrated that EGFR‐TKI dramatically altered microRNA expression profiles in NSCLC cells.^25^, ^44^ In turn, altered microRNA expression associated strongly with TKI response by modulating crucial signalling pathway.^45^ Here, we reported a mechanism by which EGFR‐mutant lung cancer cells escaped the gefitinib treatment of TKI via over‐activation of STAT3 through miR‐206 down‐regulation.

Feedback activation of STAT3 plays a prominent role in mediating drug resistance to a broad spectrum of targeted cancer therapies and chemotherapies.^46^ This feedback activation not only has been found in EGFR‐mutant NSCLC, but also in NSCLC patients with wide‐type EGFR,^47^ which may partly contribute to the intrinsic resistance and high recurrence with TKI treatment. In most cancers, STAT3 is typically active, but its activation can also occur through the influences of the microenvironment and in particular IL‐6.^48^ IL‐6 is a multi‐functional chronic inflammation cytokine, and high systemic IL‐6 expression level is associated with worse prognosis in patients with NSCLC.^49^ Except for influencing by microenvironment, it has been reported that STAT3‐dependent drug resistance could be mediated via autocrine IL‐6 production.^38^, ^50^ Hence, the autocrine IL‐6 production could exacerbate the intrinsic feedback activation of STAT3 and accelerate TKI resistance process.

STAT3 has emerged as an important regulator of gene expression, including miRNA, and in turn, several miRNAs can regulate the activity state of STAT3 in tumours.^51^ For example, STAT3 interacted directly with the promoter of miR‐21 in myeloma cells^52^ and mediated reduction of the let‐7 family via up‐regulation of Lin‐28 in breast cancer.^53^ Conversely, up‐regulation of miR‐19a/b targeted SOCS‐1, which is a negative regulator of IL‐6R/STAT3 pathway.^54^ Our study found that miR‐206 negatively regulated the activity of STAT3 by targeting intracellular IL‐6 mRNA, while STAT3 modulated miR‐206 expression level in a post‐transcriptional way.

miRNA biogenesis is a multistep process, in which pri‐miRNA is first cropped by Drosha and its cofactor DGCR8 and then cleaved by Dicer to generate approximately 22 nucleotides double‐stranded mature one.^55^ The regulation of miRNA occurs in chromatin, transcriptional and post‐transcriptional level.^56^ In the post‐transcriptional level, Chen et al have found binding of Lin‐28 to let‐7 family members could block their processing by different mechanisms at either the DROSHA or the DICER level.^57^ Michlewski et al have showed that hnRNP A1 binds to the loop of pri‐miR‐18a and induces a relaxation at the stem, creating a more favourable cleavage site for Drosha.^58^ What's more, Chen et al have reported that the expression of Dicer could affect gefitinib in human lung cancer cells.^59^ In this study, we also found a post‐transcriptional regulation of pri‐miR‐206 by binding of p‐STAT3, which resulted in reduced expression of mature miR‐206 and gefitinib resistance in EGFR‐mutant lung cancer cells.

In summary, our findings reveal a reciprocal regulation of miR‐206 and IL‐6/STAT3 pathway that mediates IL‐6‐induced gefitinib resistance in EGFR‐mutant lung cancer cells. We demonstrated that tumour microenvironment cytokine could exacerbate gefitinib resistance by regulating miRNA indirectly.

## CONFLICTS OF INTEREST

The authors confirm that there is no conflict of interest.

## AUTHOR CONTRIBUTIONS

Yanhua Yang and Tingguo Zhang conceived the study and designed the experiments. Yanhua Yang, Wei Wang and Hong Chang performed the experiments. Zenglei Han and Xinjuan Yu contributed to the data collection and analysis. Yanhua Yang wrote the manuscript. All authors read and approved the final manuscript.

## Data Availability

The data that support the findings of this study are available on request from the corresponding author. The data are not publicly available because of privacy or ethical restrictions.
