# Supplementary material for: Reciprocal regulation of miR‐206 and IL‐6/STAT3 pathway mediates IL6‐induced gefitinib resistance in EGFR‐mutant lung cancer cells
Source: J Cell Mol Med. 2019 Sep 10;23(11):7331–41. doi: 10.1111/jcmm.14592 (PMC6815809; doi:10.1111/jcmm.14592)
Supplement: Supplementary file 1 [file JCMM-23-7331-s001.docx]

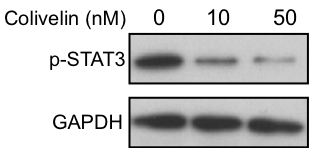


**Figure S1** Colivelin activated p-STAT3 in PC-9 cells. Cells were collected 6h after colivelin addition.


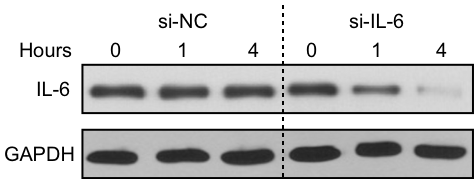


**Figure S2** Intracellular IL-6 knockdown by 50nM si-IL-6 in PC-9 cells.


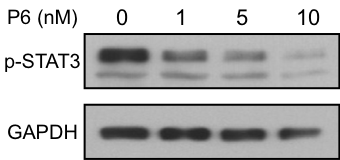


**Figure S3** P6 inhibited p-STAT3 in PC-9 cells. Cells were collected 4h after P6 addition.
