# Supplementary material for: Reciprocal regulation of miR‐206 and IL‐6/STAT3 pathway mediates IL6‐induced gefitinib resistance in EGFR‐mutant lung cancer cells
Source: J Cell Mol Med. 2019 Sep 10;23(11):7331–41. doi: 10.1111/jcmm.14592 (PMC6815809; doi:10.1111/jcmm.14592)
Supplement: Supplementary file 2 [file JCMM-23-7331-s002.docx]

**Supplementary Table 1. Clinical characteristics of patients**

| **Patient ID** | **Gender** | **Age** | **Smoker** | **Pathological Diagnosis** | **Furman Grade** | **TNM Stage** | **Treatment** | **Objective Response** | **Month of Treatment** | **EGFR Mutation** | | | |
| --- | --- | --- | --- | --- | --- | --- | --- | --- | --- | --- | --- | --- | --- |
|  |  |  |  |  |  |  |  |  |  | **19DEL** | **L858R** | **T790M** | **Others** |
| 01 | M | 54 | Y | LAD | ⅢB | T_2_N_3_M_0_ | Gefitinib | PR | 7 | √ |  |  |  |
| 02 | M | 67 | N | LAD | ⅢC | T_3_N_3_M_0_ | Gefitinib | PR | 9 |  | √ | √ |  |
| 03 | F | 45 | Y | LAD | ⅡB | T_2_N_1_M_0_ | Gefitinib | PR | 15 | √ |  |  |  |
| 04 | M | 56 | N | LAD | ⅡA | T_2_N_0_M_0_ | Gefitinib | SD | 14 |  | √ |  |  |
| 05 | M | 75 | N | LSCC | ⅡA | T_2_N_0_M_0_ | Gefitinib | SD | 11 |  |  | √ |  |
| 06 | F | 67 | Y | LSCC | ⅢC | T_4_N_3_M_0_ | Gefitinib | PR | 9 |  |  | √ |  |
| 07 | F | 46 | N | LSCC | ⅢA | T_3_N_2_M_0_ | Gefitinib | SD | 15 |  | √ |  | √ |
| 08 | M | 50 | N | LAD | IVA | T_3_N_2_M_1b_ | Gefitinib | SD | 12 | √ |  |  |  |
| 09 | F | 65 | N | LAD | ⅡA | T_2_N_0_M_0_ | Gefitinib | PR | 17 |  |  | √ |  |
| 10 | M | 54 | Y | LSCC | IVB | T_3_N_3_M_1c_ | Gefitinib | SD | 18 |  | √ |  |  |
| 11 | M | 46 | N | LAD | IVB | T_3_N_4_M_1c_ | Gefitinib | SD | 9 |  |  | √ | √ |
| 12 | M | 73 | N | LAD | ⅢA | T_2_N_2_M_0_ | Gefitinib | PR | 7 | √ |  |  |  |
| 13 | M | 46 | Y | LSCC | IVA | T_3_N_2_M_1a_ | Gefitinib | PR | 6 |  | √ |  | √ |
| 14 | F | 47 | Y | LAD | ⅡB | T_2_N_1_M_0_ | Gefitinib | SD | 12 | √ |  | √ |  |
| 15 | F | 60 | N | LAD | ⅢA | T_2_N_2_M_0_ | Gefitinib | SD | 13 |  |  | √ |  |
| 16 | M | 67 | N | LSCC | IVB | T_4_N_2_M_1c_ | Gefitinib | SD | 19 |  | √ |  | √ |
| 17 | M | 56 | N | LAD | ⅢA | T_2_N_2_M_0_ | Gefitinib | PR | 15 | √ | √ |  |  |
| 18 | F | 73 | N | LAD | IVB | T_2_N_3_M_1c_ | Gefitinib | PR | 13 | √ |  |  | √ |
| 19 | F | 47 | Y | LSCC | ⅡB | T_2_N_1_M_0_ | Gefitinib | SD | 13 |  | √ |  |  |
| 20 | F | 55 | N | LAD | ⅢA | T_2_N_2_M_0_ | Gefitinib | PR | 14 |  |  | √ |  |
| 21 | F | 47 | Y | LAD | ⅢB | T_2_N_3_M_0_ | Gefitinib | SD | 12 | √ |  |  |  |
| 22 | M | 57 | N | LAD | ⅢA | T_2_N_2_M_0_ | Gefitinib | SD | 17 |  | √ |  | √ |
| 23 | F | 58 | N | LAD | IVA | T_2_N_3_M_1a_ | Gefitinib | PR | 14 |  |  | √ |  |
| 24 | M | 72 | Y | LSCC | ⅢA | T_2_N_2_M_0_ | Gefitinib | PR | 8 |  | √ |  | √ |
| 25 | M | 41 | N | LAD | IVB | T_4_N_3_M_1c_ | Gefitinib | SD | 13 |  | √ |  |  |
| 26 | M | 43 | Y | LSCC | ⅢB | T_2_N_3_M_0_ | Gefitinib | SD | 9 | √ |  | √ |  |
| 27 | F | 47 | Y | LAD | ⅢA | T_2_N_2_M_0_ | Gefitinib | SD | 14 |  | √ |  |  |
| 28 | F | 51 | N | LSCC | IVB | T_4_N_4_M_1c_ | Gefitinib | PR | 15 |  |  | √ |  |
| 29 | F | 64 | Y | LSCC | IVA | T_3_N_3_M_1b_ | Gefitinib | PR | 19 | √ |  |  |  |
| 30 | M | 67 | N | LAD | IVA | T_3_N_4_M_1a_ | Gefitinib | SD | 21 | √ |  |  |  |
| 31 | F | 75 | N | LAD | ⅢA | T_2_N_3_M_0_ | Gefitinib | SD | 22 |  | √ |  | √ |
| 32 | M | 45 | N | LAD | IVB | T_4_N_4_M_1c_ | Gefitinib | PR | 17 |  |  | √ |  |
| 33 | F | 45 | N | LSCC | IVB | T_4_N_4_M_1b_ | Gefitinib | SD | 16 |  | √ |  |  |
| 34 | F | 46 | N | LAD | ⅡB | T_2_N_0_M_0_ | Gefitinib | SD | 8 | √ | √ |  |  |
| 36 | M | 47 | N | LAD | ⅢB | T_3_N_2_M_0_ | Gefitinib | PR | 12 |  |  | √ |  |
| 37 | F | 62 | Y | LAD | ⅢA | T_2_N_2_M_0_ | Gefitinib | PR | 14 |  | √ | √ | √ |

Abbreviation: M, Male; F, Female; Y, Yes; N, No; LAD, lung adenocarcinoma; LSCC, lung squamous cell carcinoma; TNM, Tumor/Node/Metastasis; PR, partial response; SD, stable disease
