# Supplementary material for: Reciprocal regulation of miR‐206 and IL‐6/STAT3 pathway mediates IL6‐induced gefitinib resistance in EGFR‐mutant lung cancer cells
Source: J Cell Mol Med. 2019 Sep 10;23(11):7331–41. doi: 10.1111/jcmm.14592 (PMC6815809; doi:10.1111/jcmm.14592)
Supplement: Supplementary file 3 [file JCMM-23-7331-s003.docx]

**Supplementary Table Ⅱ. The primers used for qRT-PCR and constructs sequence**

| **Gene name** | **Sequence of primers (5'-3')** |
| --- | --- |
| GAPDH F | TCAACGACCACTTTGTCAAGCTCA |
| GAPDH R | GCTGGTGGTCCAGGGGTCTTACT |
| U6 F | GATTATCGGGACCATTCCACTG |
| U6 R | GATCTGGTTCCCAATGACTGTG |
| miR-206 | UGGAAUGUAAGGAAGUGUGUGG |
| miR-206 F | TGGAATGTAAGGAAGTGTGTGG |
| miR-206 R | Universal Adaptor PCR Primer (QP016, GeneCopoeia^TM^) |
| pre-miR-206 | UGCUUCCCGAGGCCACAUGCUUCUUUAUAUCCCCAUAUGGAUUACUUUGCUAUGGAAUGUAAGGAAGUGUGUGGUUUCGGCAAGUG |
| pre-miR-206 F | CACTTGCCGAAACCACACA |
| pre-miR-206 R | Universal Adaptor PCR Primer (QP016, GeneCopoeia^TM^) |
| pri-miR-206 F | GATTTGGTCCCAAGCAGCAC |
| pri-miR-206 R | GCCCAGGCAGGAAACTCTTA |
| IL6 F | GAGAAAGGAGACATGTAACAAGAGT |
| IL6 R | GCGCAGAATGAGATGAGTTGT |
| IL6R F | CATGTGCGTCGCCAGTAGT |
| IL6R R | AGCTCAAACCGTAGTCTGTAGA |
| gp130 F | CGGACAGCTTGAACAGAATGT |
| gp130 R | ACCATCCCACTCACACCTCA |
| JAK1 F | GTAATAAAGGAGTCTGTGGTCAGC |
| JAK1 R | TGCAGAGGTAATGATGGGCATC |
| Stat3 F | CCTCAGCAGGAGGGCAGT |
| Stat3 R | CTGTGTGAGGGGTGGCAG |
| EGFR F | AGGCACGAGTAACAAGCTCAC |
| EGFR R | ATGAGGACATAACCAGCCACC |
| SOCS1 F | CACGCACTTCCGCACATTC |
| SOCS1 R | TAAGGGCGAAAAAGCAGTTCC |
| SOCS3 F | CCTGCGCCTCAAGACCTTC |
| SOCS3 R | GTCACTGCGCTCCAGTAGAA |
| PIAS3 F | CTGGGCGAATTAAAGCACATGG |
| PIAS3 R | AAAGCGTCGTCGGTAAAGCTC |
| SHP-2 F | GAACTGTGCAGATCCTACCTCT |
| SHP-2 R | TCTGGCTCTCTCGTACAAGAAA |
| Cyclin D1 F | GCTGCGAAGTGGAAACCATC |
| Cyclin D1 R | CCTCCTTCTGCACACATTTGAA |
| SOX9 F | AGCGAACGCACATCAAGAC |
| SOX9 R | CTGTAGGCGATCTGTTGGGG |
| CDK9 F | ATGGCAAAGCAGTACGACTCG |
| CDK9 R | GCAAGGCTGTAATGGGGAAC |
| TWF1 F | GACCGGCATCCAAGCAAGT |
| TWF1 R | TCCTCCAACAGGGGTAAAACA |
| CXCL11 F | GACGCTGTCTTTGCATAGGC |
| CXCL11 R | GGATTTAGGCATCGTTGTCCTTT |
| si-IL6 | ACCCAACCACAAATGCCAG |
